# Scalable, context-sensitive psychiatric assessment with large language models and brief diaries

**DOI:** 10.1017/S0033291726105261

**Published:** 2026-07-21

**Authors:** Whitney R. Ringwald, Aman Taxali, Mike Angstadt, Colin E. Vize, Chandra Sripada, Aidan G. C. Wright

**Affiliations:** 1 University of Minnesota, USA; 2 University of Michigan, USA; 3 University of Pittsburgh, USA

**Keywords:** ambulatory assessment, artificial intelligence, daily diary, hierarchical taxonomy of psychopathology, large language models, natural language processing, psychometrics

## Abstract

**Background:**

Accurate psychiatric assessment requires understanding a person’s unique experience within their psychosocial context. Clinical interviews have been the gold standard for assessment as the only methods capable of this complex task, but they are time and resource-intensive. Consequently, psychiatric assessment typically relies on patient report surveys that are decontextualized and narrow in scope. This comprehensiveness-scalability tradeoff is a major bottleneck in studying and treating psychopathology. We propose using large language models (LLMs) to score psychopathology from brief personal narratives as a low-burden, context-sensitive solution.

**Methods:**

Participants (*N* = 108) completed brief (~1 minute), freeform audio diaries daily for 2 weeks. We used six LLMs to score wide-ranging psychopathology (Internalizing, Detachment, Disinhibition, Antagonism, Anankastia) from the diary transcripts. Leveraging an array of self-report and clinical interview measures, we tested the convergent, discriminant, concurrent, and clinical validity of LLM ratings for between-person differences and within-person fluctuations in psychopathology.

**Results:**

Supporting convergent and discriminant validity, LLM ratings correlated most strongly with corresponding self-report domains at the between (average convergent *r* = .42) and within-person (*r* = .28) levels. LLM and self-report ratings had similar patterns of associations with external variables, except for Anankastia and Antagonism. Further, every LLM-rated domain related to psychopathology ascertained by clinical interview.

**Conclusions:**

Across multiple forms of validity, we showed that LLMs can assess most major forms of psychopathology from mere minutes of audio. These results support scoring open-ended narratives with LLMs as a scalable, portable method to translate idiographic diagnostic data into standardized psychiatric assessments.

Psychiatric assessment is challenging – boundaries are blurry between normality and clinical impairment, and between different diagnoses. Making these distinctions involves extracting diagnostic information from large amounts of data about a person’s unique experiences within their psychosocial context. That is, it involves listening to idiosyncratic narratives and translating them into the vocabulary of psychopathology signs and symptoms that allow us to understand a person’s impairment, compare them to others, and facilitate clinical communication. Clinical interviews are the gold standard because they have been the only methods that allow such integration and translation. However, interviews are not scalable or portable because they are time-consuming, costly, and require considerable expertise or training. Large-scale and intensive assessments, in both clinical and research contexts, have therefore often relied on patient report scales. Despite their psychometric strengths, scales are rarely tailored to the individual’s issues, lack contextual information, and are narrow in scope. This tradeoff between assessments that are individualized and context-sensitive or scalable and portable has limited our knowledge of psychopathology.

Natural language processing offers a solution to this tradeoff by automating psychopathology assessment from personal narratives. An ability to extract information from natural language is important because a person’s description of themselves, their circumstances, and their problems – in their own words – is arguably the richest source of diagnostic information we have (Pennebaker, Mehl, & Niederhoffer, 2003). However, for decades, automated language processing alternatives to resource-intensive human raters were limited to simple metrics like word counts. Word counts ignore context and cannot capture the nuance needed to assess psychopathology. Newly introduced transformer-based language models are completely different. Transformer-based models are trained on massive amounts of natural language data to learn language patterns. Then, unlike older models that process discrete, decontextualized words, transformer-based models process sequences of words, considering their interrelationships. Owing to this technological advance, these newer models encode the meaning of words within the broader *context* of the narrative (Eichstaedt et al., 2021).

Accounting for context in natural language analysis offers unprecedented capabilities for assessing psychopathology (Kjell, Kjell, & Schwartz, 2024b). This is because symptoms are often implied rather than stated. For example, the meaning implied by a sentence like ‘I keep doing things I’m supposed to enjoy, but nothing lands anymore’ is the experience of anhedonia even though a specific symptom is not named. Perhaps more fundamentally, discerning normality from clinical impairment hinges on the context of behavior (Hopwood, Wright, & Bleidorn, 2022). A phrase like ‘I felt stressed’ could indicate impairment if the stress is in response to a minor hassle and becomes overwhelming but could be normal if it was manageable stress in anticipation of a high-stakes job interview. Likewise, context is necessary to distinguish different kinds of psychopathology. For example, distinct forms of psychopathology would be implied by *feeling stressed* about social evaluation versus signs of physical illness. Transformer-based models, in principle, can make such nuanced distinctions when analyzing text. Accordingly, these models can then compare rich, diagnostic information detected in text to its trained knowledge base and predict the kind and severity of psychopathology evident (if any). In essence, transformer-based models could solve the scalability problem by performing functions of trained human raters in seconds or minutes rather than the days or months needed to train raters and hand-code texts.

A small but growing literature supports the ability of transformer-based models to assess psychopathology from natural language. One approach has been to fine-tune models to predict specific diagnoses. Results show these bespoke models perform well – for example, depression scored from open-ended text about symptoms converges with self-reports (*r* = .83) (Gu, Kjell, Schwartz, & Kjell, 2025), and suicidal ideation scored from Reddit posts align with clinician ratings (AUC = .83) (Gaur et al., 2021). A downside of fine-tuned models is that they require substantial time and resources to create. Development entails training models in very large samples and technical expertise beyond that of a typical clinical scientist or practitioner interested in using them. Moreover, a new model must be developed for each psychopathology construct one wishes to assess, imposing major practical constraints on the breadth of assessment possible with this method. Thus, fine-tuned language models address some barriers to scalability while introducing new ones.

A more scalable approach may be to assess psychopathology with commercially available large language models (LLMs) (e.g., ChatGPT) (Brickman, Gupta, & Oltmanns, 2025). LLMs are trained on billions of text documents from diverse internet-based sources, including scientific articles, digitized books, news articles, and social media. This massive training dataset likely contains information relevant to predicting virtually any form of psychopathology that’s been written about in human history. Because of their pretraining, LLMs can assess psychopathology from language without the need for fine-tuning, referred to as *zero-shot*. Indeed, results of studies using zero-shot approaches are promising. For example, it’s been shown that zero-shot ratings of depression and posttraumatic stress disorder from diagnostic interviews predict clinician ratings and self-reports with model accuracies ranging from .73 to .94 (Narita et al., 2025; Ohse et al., 2024), and LLM ratings of depression and suicidality from social media posts achieve accuracies up to .83 in predicting expert labels (Lamichhane, 2023; Xu et al., 2024).

An especially attractive feature of zero-shot scoring with LLMs is that the cost, researcher effort, and participant burden of assessing each additional construct are relatively minor. Consequently, this approach can minimize constraints inherent to nearly all other measurement tools on the breadth of psychopathology that can be assessed for a given person. The ability to score many constructs from a single language sample would be especially impactful for intensive longitudinal assessments where participant burden is of utmost concern. Here, assessment of a given construct is typically limited to one self-report item or a handful at best. Regardless of assessment setting, rather than confining our view of human experience to a list of generic, close-ended survey items out of practical necessity, zero-shot scoring of natural language could break free of these confines to systematize personalized, context-sensitive, and comprehensive psychopathology assessment at scale.

For zero-shot LLM assessments to be scalable and portable (i.e., measurable in the flow of daily life), psychopathology must be detectable in brief text samples – yet this has not been well-established. Performance of zero-shot assessments has mostly been studied using lengthy language samples that require significant effort to collect, such as clinical interviews and social media histories. If collecting the requisite language data is costly and burdensome, the efficiency afforded by LLMs on the backend is essentially moot. A small number of recent studies suggest that psychiatric symptoms can, in fact, be detected in brief text samples. For example, one study found strong agreement between LLM ratings and expert ratings of depression from participant’s short, open-ended descriptions of their depression (*r* = .81), and the LLM ratings converged with self-reports (*r* = .46) (Ganesan et al., 2024). While the convergent validity is impressive, responses to a prompt focused on specific symptoms narrow the range of information available to assess other constructs.

Addressing the comprehensiveness-scalability tradeoff then requires language samples that are not only brief, but that also contain information needed to validly assess multiple forms of psychopathology. This is a tall order. Yet the solution may be simple: *unconstrained narratives*. Responses to minimally directed, open-ended prompts like ‘talk about the most significant event in your day’ may be an efficient way to sample the most relevant aspects of a person’s current psychological functioning. Without much direction, people will talk about personally salient experiences, including the relationships, events, and thoughts that matter most to them. This naturally occurring information selection process may serve as a filter, resulting in a diagnostically dense sample of language. There is some evidence supporting this possibility. Studies using zero-shot scoring with LLMs have shown depression severity can be assessed from aggregated responses to free-text daily diary entries (Shin et al., 2024) and routine voicemails left at a clinic (Kim et al., 2025), and posttraumatic stress symptom severity can be assessed from brief (~8 minute) responses to prompts about general life circumstances (Kjell et al., 2024a).

Despite initial evidence that zero-shot LLM ratings can be a scalable and portable approach to assess psychopathology, there are substantial gaps in knowledge about the scope of application and validity of this method. First off, studies have only assessed internalizing pathology from a person’s natural language with LLMs, so it is unknown what other forms of psychopathology – if any – can be detected in unconstrained narratives. Indeed, the general distress definitive of internalizing problems could make it an especially ‘easy target’ to detect in natural language, whereas kinds of psychopathology that are marked less by distress and more by subtle, context-specific thoughts and behavior (e.g., disinhibition, antagonism) may be less apparent without targeted prompting. Thus, one of the most potentially revolutionary features of zero-shot LLMs – an ability to assess wide-ranging psychopathology from a single brief language sample – has yet to be established.

Basic questions about the validity of zero-shot LLM assessments have also not been tested. Validating any new measure is important, but the standards should be even higher when introducing an entirely novel method such as LLM ratings, but studies to date have only tested their *convergent validity*. Beyond convergence, it is necessary to also establish that zero-shot LLM scores can (1) distinguish different forms of psychopathology (i.e., *discriminant validity*), (2) relate to external variables in expected ways (i.e., *concurrent validity*), and (3) pick up on clinically relevant signs and symptoms (i.e., *clinical validity*). Without meeting these standards of validity, LLM assessments will be of minimal use in clinical psychological research or practice.

Finally, although measuring psychopathology from brief narratives with LLMs could in principle be the holy grail of intensive longitudinal assessment by enabling comprehensive, low-burden monitoring, this has not been investigated. Results from two intensive longitudinal studies in the same sample hint at this possibility, finding negative affect and activation scored from free-text responses about daily events track momentary self-reported affect (Fisher et al., 2025a, 2025b). But with LLM’s advanced contextual reasoning, they may be capable of tracking much more than just emotional arousal and valence. To move from principle to reality, a first step is validating LLM ratings for both overall psychopathology and for tracking subtle, day-to-day symptom or behavior changes.

## Present study

This present study aimed to establish the breadth of psychopathology that can be assessed by LLMs from brief, unconstrained narratives. We studied a sample of participants who completed 1-minute diaries talking about the day’s events, each day, for about two weeks. Our previous work in this sample showed LLMs can score all Big Five personality traits from the daily diaries, suggesting most variation in normal-range functioning can be detected in these narratives (Wright, Ringwald, Vize, et al., 2025) – the present study builds on this precedent to ask whether most forms of *maladaptive* functioning are also evident. To that end, we employed six different LLMs to score major domains of psychopathology: Internalizing, Detachment, Disinhibition, Antagonism, and Anankastia (i.e., pathological constraint). We then capitalized on an array of self-report and clinical interview measures to evaluate the convergent, discriminant, concurrent, and clinical validity of LLM ratings. Validity was tested for assessing both between-person differences and within-person fluctuations in psychopathology.

## Methods

Supplementary Materials are on the Open Science Framework: https://osf.io/tqsz5/

### Participants and procedures

Participants were community members recruited through posted flyers and an online clinical research registry. Screening was used to recruit a sample balanced on sex and reported mental health treatment history (current/past treatment or not). To be eligible, participants had to be between the ages 18 and 40 and not receiving treatment for a psychotic disorder.

Study procedures involved an in-person assessment followed by an ambulatory assessment protocol. At the baseline assessment, participants completed a clinical interview and self-report questionnaires. Length of the ambulatory protocol was 21 days for the initial 37 participants and was then reduced to 14 days for administrative reasons. Surveys and video diaries were completed daily through a smartphone application. The video diary protocol was discontinued partway through the study to accommodate other study procedures.

Of the 311 total participants, the present study included all participants who enrolled in the video diary protocol (*N* = 108). No other exclusions were applied. Included participants were 53% female with a mean age of 28.3 (SD = 6.38). On average, participants completed 14.9 (SD = 5.32) daily video diaries.

### Measures

#### Daily self-report psychopathology

Daily psychopathology was self-reported at the end of each day with an 81-item inventory validated for ambulatory assessment (Wright, Ringwald, & Zimmermann, 2025). Each item included the stem ‘Over the past 24 hours…’ followed by a statement reflecting a daily manifestation of psychopathology (e.g., ‘I acted aggressively toward someone,’ ‘I worried about being abandoned’). Items were rated on a 101-point slider scale from 0 (not at all) to 100 (very much), then averaged for scores of daily Negative Affectivity, Detachment, Antagonism, Disinhibition, and Anankastia.

#### Daily LLM-rated psychopathology

Daily psychopathology was scored with LLMs from daily diary entries. Participants responded to the prompt, ‘*Please think of the most significant event in your day today. Use the video to briefly (1 min) describe: what happened, who was there, how you behaved, and how you felt during the event.*’ The average per-video word count was 120.2 (SD = 57.7). Audio from the videos was transcribed using Whisper (Radford et al., 2022), and LLMs scored the resulting text. LLM ratings were obtained using a zero-shot approach validated in our previous work (Wright et al., 2025). We employed six state-of-the-art, commercially available LLMs: GPT-5, Claude-Sonnet-4, Gemini-2.5-Flash, Grok-3, Llama-4-Maverick, and Qwen3-235B. We used the following scoring prompt:

*Your task is to assess the participant’s level of {domain} based on their daily diary entry describing the most significant event that occurred during their day.*

*Respond as if you are the participant, using their emotional tone and inner reactions to guide your answer.*

*Pay attention to signs of {signs}. Use contextual reasoning to determine how strongly the participant likely experienced or expressed {domain}.*

Domains were rated from 0 to 100. Scores produced by the LLMs were averaged because we have found that this provides a more robust score estimate than individual LLMs (Wright et al., 2025). Seed and temperature were set to zero to ensure score consistency. Average split-half reliability for the LLM scores was .70. Complete scoring procedure (Supplementary Table S1) and reliability results are in the Supplementary Materials (Supplementary Table S2).

#### Baseline interview-rated psychopathology

Clinical validity was assessed vis-à-vis psychopathology ascertained by the baseline clinical interview measures. Interviews were conducted by clinical psychology doctoral trainees. Negative Affectivity, Detachment, Antagonism, Disinhibition, and Anankastia were rated using data gathered by the Structured Interview for *DSM-IV* Personality (Pfohl, Blum, & Zimmerman, 1997; Ringwald, Woods, & Wright, 2024). Dimensional scores were also created for internalizing (major depressive disorder, generalized anxiety disorder, obsessive compulsive disorder, and social anxiety disorder symptom sum), harmful substance use (alcohol and substance use disorder symptom sum), and personality pathology (all personality disorder symptom sum) using ratings on the Structured Clinical Interview for *DSM-5* (First, Williams, Karg, & Spitzer, 2016).

#### Criterion variables

A total of 7 within-person variables and 48 between-person variables were used to evaluate concurrent validity. From the baseline assessment, we used measures of interpersonal problems, adult attachment style, narcissism, and normal-range personality traits. From the ambulatory assessment protocol, we created daily scores using momentary ratings of positive and negative affect, impulsivity, perceived stress severity, and stressor occurrence. Additional measures information is in Supplementary Table S3.

### Analysis plan

Analyses used multilevel structural equation models (MSEMs) with Bayes estimation conducted in Mplus (Version 9) (Muthén & Muthén, 2025). MSEM handles the nested structure of data (i.e., days within participants) by decomposing repeated measures into within- and between-person latent variables. The between-person latent variables are random intercepts representing a person’s average levels of daily variables (e.g., average daily disinhibition). Baseline variables are also modeled at the between-person level (e.g., interview-rated disinhibition). The within-person latent variables reflect fluctuations in the daily variables from a person’s average level on a given day (e.g., how much more disinhibited a person is than their usual). These models allowed us to examine the validity of LLM ratings to assess overall levels of psychopathology (between-person level) and track day-to-day changes in psychopathology (within-person level). All available data were used regardless of how many days the participant was in the study. MSEM accommodates varying days per person by treating the unbalanced structure as a missing data problem and using data augmentation to produce precision-weighted estimates of the random effects.

*Convergent and discriminant validity* were examined by estimating correlations between self-reported daily psychopathology and LLM-rated daily psychopathology at the within- and between-person levels.

*Concurrent validity* was evaluated by indexing the similarity of the nomological nets for LLM-rated daily psychopathology and self-reported daily psychopathology. To do this, we first estimated correlations between LLM-rated and self-reported daily psychopathology with external variables at the within- and between-person levels. We then quantified their nomological similarity by calculating the correlation between the LLM and self-report profiles of associations with external variables. This profile correlation between LLM and self-report scales indexes how similar the two scales are in terms of capturing associations with external variables. Profile correlations ≥.85 indicate that the LLM and self-report scales are measuring very similar constructs (Lorenzo-Seva & Ten Berge, 2006).

*Clinical validity* was tested vis-à-vis correlations with psychopathology ascertained by clinical interview.

## Results

### Convergent and discriminant validity

**Figure 1.**
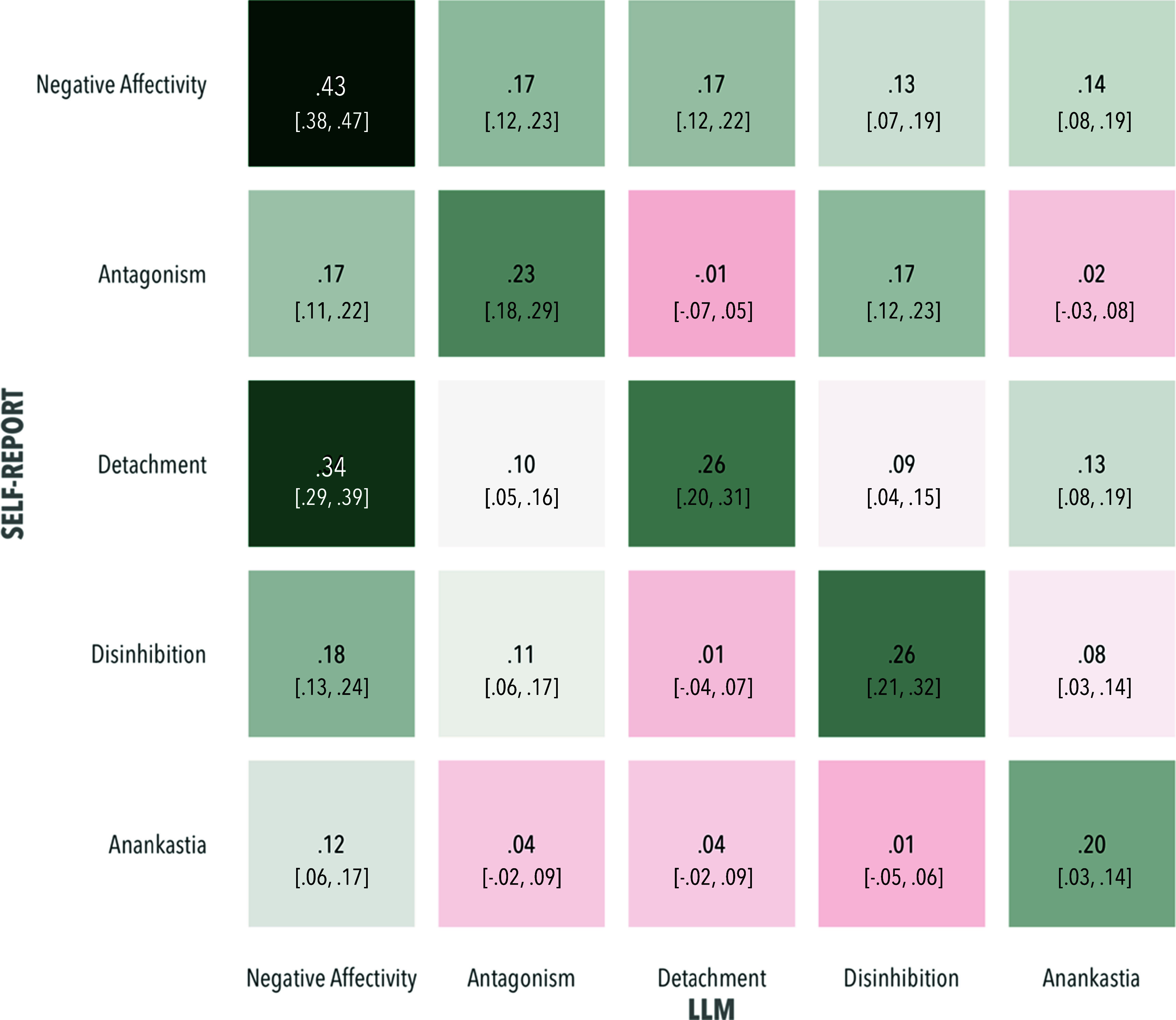
Within-person correlations between LLM ratings and self-reports of psychopathology. *Note:* LLM and self-report psychopathology reflect daily fluctuations from a person’s average levels. Credibility intervals are in parentheses. Figure 1. long description.

**Figure 2.**
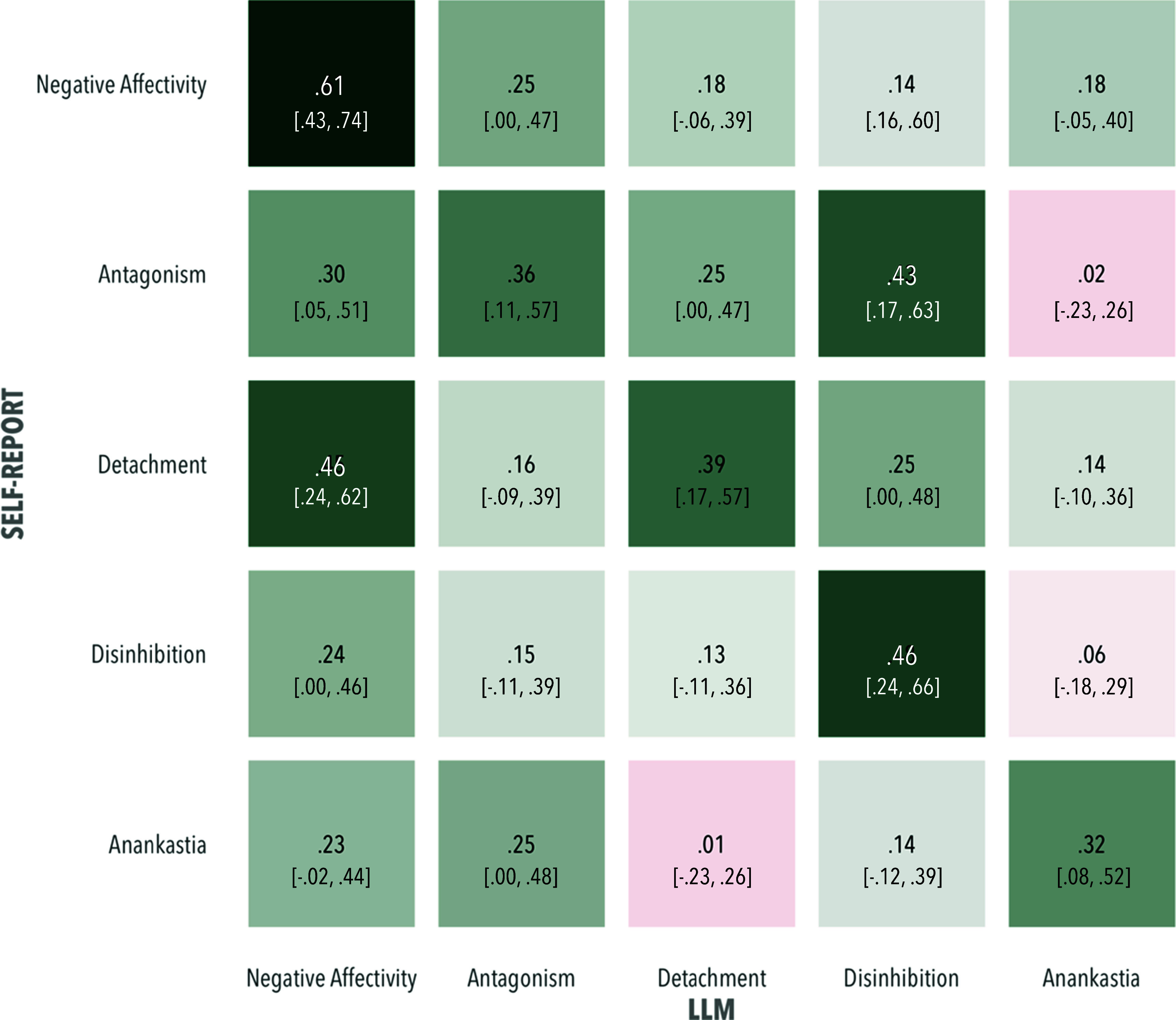
Between-person correlations between LLM ratings and self-reports of psychopathology. *Note:* LLM and self-report psychopathology reflect averages estimated from daily ratings. Credibility intervals are in parentheses. Figure 2. long description.

Multilevel correlations between LLM- and self-report ratings are in Figures 1 and 2. Model-specific results are in Supplementary Table S4a,b. At both within- and between-person levels, the strongest correlations were between corresponding LLM-rated and self-report domains. The average convergent correlation was .28 within-person (range = .20–.43) and .42 between-person (range = .32–.61), with the strongest convergence for negative affectivity at both levels.

At the between-person level, we additionally examined convergence of LLM ratings with baseline measures of the five domains, assessed by the Comprehensive Assessment of Traits Relevant to Personality Disorder (Ringwald et al., 2023; measure information in Supplementary Table S2). Overall, convergence between LLM ratings and the baseline measure was somewhat weaker, with an average convergent correlation of .28, and there were several cases in which off-diagonal correlations (i.e., nonconvergent) were strongest. Notably, correlations between average daily self-reports and the baseline self-report also had multiple strong off-diagonal correlations, suggesting poor discriminant validity is a property of the methods rather than a problem specific to the LLM ratings. Full results reported in Supplementary Table S4a,b.

### Concurrent validity

Profile correlations indexing nomological net similarity are in Table 1. The nomological relationships were nearly identical for LLM-rated and self-reported daily psychopathology at the within-person level (average profile *r* = .95). There was less nomological similarity overall at the between-person level, and notable variability in similarity across domains. Negative Affectivity was the most similar, and the most dissimilar were Antagonism and Anankastia. The two largest discrepancies in correlations with external criterion (>.25 difference) were for Antagonism and Anankastia. LLM-rated daily Antagonism was generally less pathological (e.g., more agreeable, less narcissistic, less impulsive) than self-report Antagonism. LLM-rated daily Anankastia reflected less extraversion (e.g., less positive affect, lower energy) and less antagonism (e.g., less narcissistic, more agreeable) than self-report Anankastia. Full results are in Supplementary Table S6a,b.

**Table 1.** Nomological net similarity between LLM-rated psychopathology and self-report psychopathology Table 1. long description.

Psychopathology domain	Within-person profile similarity	Between-person profile similarity
Negative affectivity	.97	.88
Antagonism	.98	.60
Detachment	.93	.82
Disinhibition	.91	.78
Anankastia	.94	.38

*Note:* Coefficients represent correlations between the profile of associations for an LLM-rated and self-rated psychopathology domain and criterion variables. There were 7 criterion variables for within-person nomological nets and 48 variables for between-person nomological nets.

### Clinical validity

**Figure 3.**
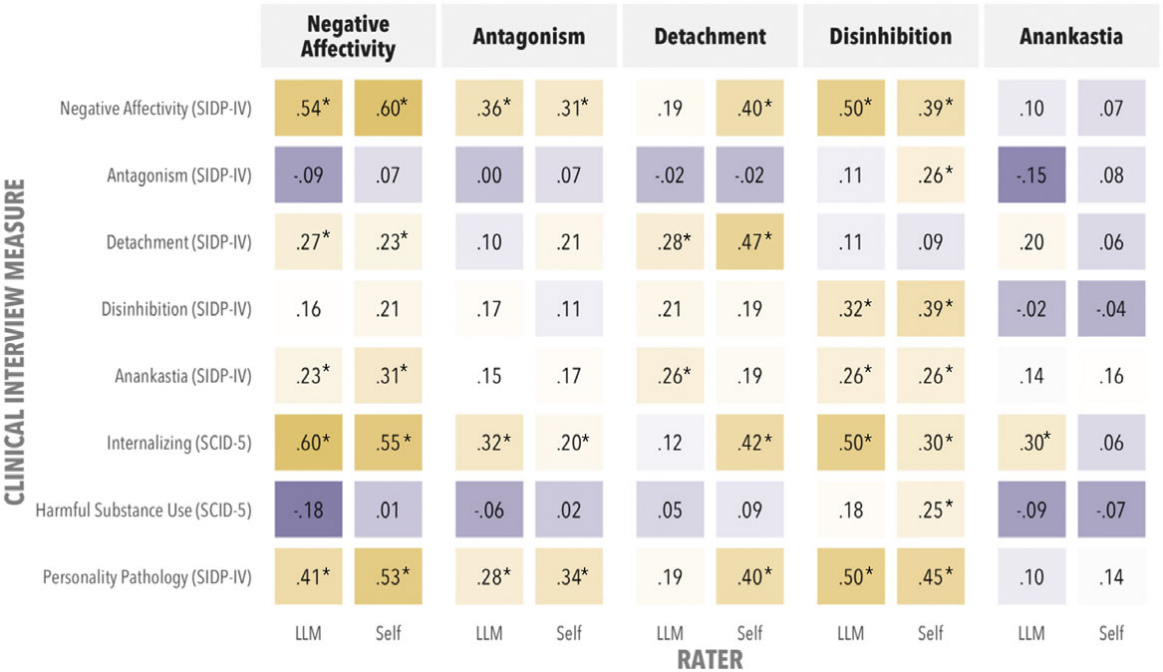
Correlations between LLM-rated and self-reported psychopathology and interview-rated psychopathology. *Note:* SIDP-IV, Structured Interview for *DSM-IV* Personality; SCID-5, Structured Clinical Interview for *DSM-5* Axis I Disorders. LLM and self-report psychopathology reflect averages estimated from daily ratings. * = credibility interval does not contain zero. Figure 3. long description.

Correlations with psychopathology ascertained by clinical interview are in Figure 3. Every LLM-rated domain related to interview measures, and most associations were as expected. LLM-ratings of daily Negative Affectivity (*r* = .54), Detachment (*r* = .28), and Disinhibition (*r* = .32) were each significantly associated with their interview-based counterparts. Negative Affectivity was further correlated with interview-based Detachment (*r* = .27), Anankastia (*r* = .23), internalizing symptoms (*r* = .60), and personality pathology (*r* = .41). Detachment was further correlated with interview-based Anankastia (*r* = .26). Disinhibition was correlated with interview-based Negative Affectivity (*r* = .50), internalizing symptoms (*r* = .50), and personality pathology (*r* = .50). Contrary to expectations, Disinhibition was not significantly correlated with harmful substance use (*r* = .18). Also unexpected is that Disinhibition was positively correlated with interview-based Anankastia (*r* = .26), though this correlation was also found for self-report Disinhibition (*r* = .28).

LLM-ratings of Antagonism and Anankastia did not correlate with their interview-based counterparts (*rs* < .15). Antagonism did, however, correlate with interview-based Negative Affectivity (*r* = .36), internalizing symptoms (*r* = .32), and personality pathology (*r* = .28) and Anankastia with internalizing symptoms (*r* = .30). For context, self-reported daily Antagonism and Anankastia also did not correlate with their interview-based counterparts (*r*s < .12), suggesting again the lack of convergence is a property of the methods rather than a shortcoming specific to LLM ratings. Further, self-report daily Anankastia did not relate to any interview variables, meaning only LLM-rated Anankastia showed evidence of clinical validity.

### Exploratory analysis of LLM rationale for scores

To gain insight into information used by LLMs to rate psychopathology, we prompted ChatGPT-5 (the best performing model; see Supplementary Table S4a,b) to provide rationales and exemplar quotes for high and low scorers for each domain. Rationales are not direct evidence for how ChatGPT-5 made ratings but are better understood as ‘post-hoc’ explanations generated by the model that can aid interpretability (Lipton, 2016). These rationales also may not completely represent the ‘logic’ of the aggregated scores we used. Select results are in Table 2.

**Table 2.** Rationales and transcript quotes selected by ChatGPT-5 to support its ratings Table 2. long description.

	High		Low	
	Quote	Rationale	Quote	Rationale
Negative affectivity	*I was upbeat and friendly, and everything was good. But as soon as it ended, and I’m back home now, I kind of feel a little bit depressed.*	A quick shift from positive to ‘a little bit depressed’ after a good event shows emotional lability toward negative mood.	*The baby was screaming, she was tired, and in her car seat, that was kind of unpleasant*	Brief irritation/stress is acknowledged due to the baby’s crying, but it is framed as mildly unpleasant rather than intensely distressing.
	*I went to the liquor store and got a couple bottles of beer and kind of went down to the river and just kind of drank them by myself… an example of me needing to be by myself and kind of feeling not great about… about my life.*	Avoidant coping, including solitary drinking when feeling bad, indicates difficulty tolerating distress and a tendency to withdraw.	*I got the keys to move into my new place, so I’ll be moving in tomorrow, I’m excited to move*	A potentially stressful life event (moving) is framed with enthusiasm rather than apprehension or dread.
Antagonism	*I had moments where I wanted to choke somebody.*	Hostile impulses surface under stress, suggesting irritability with aggressive ideation.	*I did spend a little bit too much time helping him when I could have probably gotten more help studying on my own.*	Consistently prosocial and empathic behavior; the diarist helps a less-prepared friend even at some personal cost, indicating low antagonism.
	*First, I acted on impulse, and I wasn’t trying to hear it, I was trying to have everything just ran my way, done my way.*	She shows entitlement and a need for control, initially refusing others’ input during conflict with her mother.	*I’m just a socially awkward and anxious person, so it was a little bit embarrassing*	Self-effacing and anxious self-view rather than grandiosity or entitlement.
Detachment	*I had to force myself to play it, I just could not get any enjoyment out of it…. I just kind of push through like it’s something on a checklist…. I did not feel much of anything out of it.*	Clear anhedonia: pushing through an activity without enjoyment and reporting blunted affect toward it.	*My husband was there with me though. He stayed with me the whole time, which was awesome. So it was nice to be with somebody.*	Seeks and values closeness and support (with spouse, coworkers, friends), inconsistent with detachment or intimacy avoidance.
	*Absolutely nothing. There was nothing significant in my day today… I just did not do anything today. I just did not feel up to doing anything.*	Several days of staying in, low activity, and lack of motivation suggest mild social withdrawal, though often attributed to weather or sleep issues rather than a pervasive emotional distancing.	*Very enjoyable and low-key, just spending time together and talking and hanging out.*	Consistent closeness and physical/emotional intimacy with her partner indicate low detachment.
Disinhibition	*So I came out to a silent rave… and decided that this would be a good idea. Um, it’s exciting, but, I do not know, I maybe should have slept.*	Sensation seeking and difficulty delaying gratification are suggested by choosing an exciting event over sleep, indicating poor planning and short-term reward focus.	*We had food, and we drank, and it was a good time.*	Social events include drinking, but there is no indication of excess, risky behavior, or disregard for consequences; the tone is moderate and controlled.
	*Everything that’s happened to the car so far, like tickets and scratches and towings, have all been my fault.*	Repeated mishaps with someone else’s car point to irresponsibility/poor planning and possible careless decision-making, a facet of disinhibition.	*Probably the most significant event I engaged in today was meeting with a professor about research stuff.*	Entries center on planned, structured commitments (interviews, meetings), which run counter to impulsivity or poor planning
Anankastia	*I pushed myself really hard working on it. My neck has been hurting working on the computer for so long. When I finished it wasn’t really exhilarating, but more just able to move on to the next thing, the next stressor, so it’s not exactly as relaxing as it should be.*	Overconscientious, driven work style with difficulty relaxing after completion suggests an anankastic focus on duty and next tasks rather than satisfaction.	*We’re still up because we took a nap… we just get to rock and have fun.*	Vacation behavior appears spontaneous and unstructured, not detail-focused or rule-bound.
	*Usually I try to start studying about a week before the test. I basically just stopped. Stopped what I was doing, and got to work right away.*	Preference for structured planning and timely preparation; immediate corrective action when schedule tracking falls short.	*It’s another late night tonight, obviously, so good going me. Whatever. Just to try and clear my head a little bit. It was good. It was good, I think. I think it helped. I guess we’ll see.*	Attitude toward routines and slip-ups is relaxed, not over-conscientious or rule-bound; there is little sign of preoccupation with order or fear of errors.

Overall, the rationales appear face valid, suggesting that ChatGPT-5 differentiated pathology from normative behavior by using broader contextual information and implied dysfunction rather than overt symptoms. For example, the absence of apprehension in anticipation of an often-stressful event (moving) was cited as evidence of low Negative Affectivity. Some rationales suggested that ratings were made with respect to inferred mechanisms. For example, solitary drinking when down was cited as evidence for avoidant coping related to Negative Affectivity rather than impulsive-driven substance use associated with Disinhibition.

## Discussion

This study evaluated zero-shot scoring of brief, open-ended narratives with LLMs as a scalable, portable method to translate idiographic diagnostic data into standardized assessments of psychopathology. Across multiple tests of validity, we showed that this method can assess domains covering most major forms of psychopathology from mere minutes of audio. Results support the potential for this method to revolutionize psychiatric assessment while also highlighting open questions for future research.

We found LLM ratings converge with self-reports, both for assessing overall levels of psychopathology and tracking day-to-day changes in behavior. To put these results in perspective, the convergence we found between LLM ratings and self-reports (average *r* = .42) was nearly identical to meta-analytic estimates for self-informant agreement of psychopathology (*r*s = .43–.45) (Achenbach, Krukowski, Dumenci, & Ivanova, 2005; Oltmanns & Oltmanns, 2019). In other words, LLM scores made from brief narratives were as aligned with a person’s self-views as are judgments made by close friends, family, and skilled clinicians.

Unlike previous work with zero-shot LLMs that has exclusively focused on internalizing, we were able to establish the breadth and specificity of psychopathology that can be detected with this method. For every domain, at both between- and within-person levels, on-target correlations with self-reports were stronger than off-target correlations. This evidence for discriminant validity suggests that LLMs can pick up cues specific to each domain in open-ended narratives, not just general distress.

We further evaluated whether LLM and self-reports reflect roughly the same aspects of a given domain by comparing each method’s relation to external criteria. At the within-person level, LLM and self-reports had almost identical nomological nets, suggesting that what people reveal when freely talking about their day largely captures the same information about daily functioning as explicit symptom ratings. The nomological nets were generally similar for assessing overall psychopathology as well, though there was notably less similarity in the Antagonism and Anankastia constructs.

Differences in what’s reflected by LLMs versus self-report measures can be understood by considering information available to each ‘rater.’ LLMs have access to vast domain knowledge applied to information people choose to disclose in the diary (implicitly or explicitly), whereas the individual has access to vast amounts of personal experience applied to specific, researcher-determined questions. Turning then to differences in antagonism, we found self-ratings reflected more immoral, distress-laden, impulsive tendencies than LLM ratings. This suggests that people do not spontaneously talk about these kinds of behavior when talking about their day but will disclose when asked directly. For Anankastia, LLM ratings reflected the core features of emotional constraint, risk aversion, and rigid morality more so than self-ratings. The LLMs stronger associations with theoretically expected variables, despite scoring the same domains as self-reports, may stem from the LLM’s ability to discern target constructs using contextual information.

Finally, we evaluated the clinical validity of LLM ratings by examining whether they correspond to the best available method for psychiatric assessment – clinical interviews. Convergence with analogous interview measures was supported for LLM-rated domains except for Antagonism and Anankastia, and all domains generally related to *DSM* symptom counts as expected. Notably, inconsistent convergence with the analogous interview-based measure appears to be a more general methodology issue, not specific to LLM ratings, as self-reports showed the same patterns of nonconvergence. In fact, LLM and self-report ratings had largely similar associations with interview-based psychopathology. The biggest difference was for Anankastia; mirroring the nomological net results, LLM ratings of Anankastia actually had stronger associations with interview measures (albeit not with interview-rated Anankastia). Together, we provide the first evidence that zero-shot ratings of psychopathology from open-ended narratives predict clinical diagnoses, with performance on-par with the current fieldwide standard of self-reports.

### Implications for measurement, clinical practice, and theory

Our results support the potential for LLMs to transcend the assessment comprehensiveness-scalability tradeoff that’s stalled scientific progress. The most profound impact of this method could be in study designs with high administrative burden, such as large-scale databases (e.g., UK Biobank) and intensive longitudinal studies. With sufficient validation of zero-shot ratings, it may be possible for brief text samples to supplant dozens of self-reports with interview-quality assessments. This would free up resources and provide higher quality, more psychometrically sound clinical phenotypes that could improve research on population health, etiology, maintenance mechanisms, and biomarker discovery. Further, LLMs could be used to study psychopathology in any rich archival datasets with audio recordings or free-text responses, even if psychopathology wasn’t assessed.

In clinical settings, LLMs could enable recommended assessment-based practices that are rarely implemented in the real world due to high patient/administrative burden (Krist et al., 2014; Lewis et al., 2019). In particular, our finding that psychopathology can be measured from undirected language presents novel solutions to this burden problem by using data already collected in the clinical workflow. For example, LLMs could make measurement-based care (i.e., symptom assessments at each clinical encounter) more feasible by assessing psychopathology from dialog in therapy sessions. LLMs could also facilitate comprehensive mental health screening in primary care by detecting psychopathology from patient–provider conversations in the clinic or voicemail messages. LLM-based assessment tools like these could promote early detection and treatment of psychopathology by enhancing clinical judgment. However, many open questions about LLMs need to be addressed before clinical implementation, which we turn to next.

### Open questions and future directions

As the first study to evaluate the validity of zero-shot ratings of broadband psychopathology, our aim was to establish whether this method is at least comparable to standard assessment practices (i.e., self-report). Our findings show LLM ratings largely clear this bar. Moreover, because the LLM ratings were made with a one-minute video rather than a relatively lengthy 81-item survey, the LLM ratings are arguably superior when considering the tradeoff of validity and burden. This lays a necessary foundation for continued investigation into whether this method can eventually outperform self-reports. We next discuss important research directions to this end.

Like any new measurement tool, generalizability and bias need to be evaluated. Our findings are specific to brief, smartphone-based narratives completed by English-speaking adults in a research setting. This method of data collection and scoring may not produce the same results in samples with different demographics, who are non-English speaking, or in more acute clinical populations. Moreover, we were unable to test bias across demographic and cultural groups in our sample. Bias is a concern that applies to any diagnostic measure, including LLMs. LLMs can perpetuate biases present in the training data, meaning its ratings could be influenced by broad societal stereotypes, norms, and values related to psychopathology (e.g., Zack et al., 2024). Moreover, LLMs are trained on predominantly English text, which could mean inaccuracies when analyzing language of non-English speakers, pathologizing cultural normative behavior, or missing culture-specific manifestations of psychopathology (Timmons et al., 2023). Of course, because LLMs simply reflect existing human biases, gold standard interview measures suffer the same limitations. What differs is how bias can be mitigated – with LLMs, proposed solutions include, for example, targeted prompting strategies or ‘few-shot’ model training that involves exposing the model to underrepresented exemplars so that it learns culture-specific information (Shah, Schwartz, & Hovy, 2020). In principle, LLMs could eventually be less biased than interviewers because controls can be implemented in a consistent, standardized way.

Another key consideration for zero-shot LLM ratings is the source of language data. It is logical to assume that data elicited with different formats (e.g., prompted versus naturalistic conversation) and in different settings (e.g., crisis calls versus routine patient–provider interactions) will produce different kinds of information for LLMs to score. Although very little work has directly compared LLM ratings derived from different sources, a meta-analysis showing that the predictive accuracy of language-based depression assessments varies by text source adds some empirical support for this idea (Fisher et al., 2026). Thus, at minimum, the reliability and validity of LLM scores should be tested for each type of format and setting. More broadly, research on what language data sources is optimal (or adequate) for scoring a given construct will produce vital scientific and practical knowledge for using psychiatric assessment with LLMs.

Other methodological considerations specific to LLMs include the stability and validity of scores across model updates, ensuring sensitive information is not leaked into the training data (especially in clinical settings), and difficulty preventing or solving performance issues due to being a ‘black-box’ (Obradovich et al., 2024). Generally, the sheer unprecedentedness of LLM technology demands a cautious approach to implementation and human oversight for the foreseeable future. At the same time, our results add to growing evidence that it is exactly this unprecedentedness that poises LLMs as a method to overcome the comprehensiveness-scalability tradeoff that has plagued clinical science and practice.

## Supporting information

10.1017/S0033291726105261.sm001 Ringwald et al. supplementary material Ringwald et al. supplementary material

## Supplementary material

The supplementary material for this article can be found at http://doi.org/10.1017/S0033291726105261.

## Long descriptions

### Figure 1. Long description

The matrix features five psychopathology domains on both axes: Negative Affectivity, Antagonism, Detachment, Disinhibition, and Anankastia. Each cell contains a correlation coefficient and a credibility interval in brackets. Darker green indicates higher positive correlation, while light pink indicates near-zero or slightly negative values.

* Row 1, Self-Report Negative Affectivity: Correlations with L L M ratings are .43 for Negative Affectivity, .17 for Antagonism, .17 for Detachment, .13 for Disinhibition, and .14 for Anankastia.

* Row 2, Self-Report Antagonism: Correlations with L L M ratings are .17 for Negative Affectivity, .23 for Antagonism, -.01 for Detachment, .17 for Disinhibition, and .02 for Anankastia.

* Row 3, Self-Report Detachment: Correlations with L L M ratings are .34 for Negative Affectivity, .10 for Antagonism, .26 for Detachment, .09 for Disinhibition, and .13 for Anankastia.

* Row 4, Self-Report Disinhibition: Correlations with L L M ratings are .18 for Negative Affectivity, .11 for Antagonism, .01 for Detachment, .26 for Disinhibition, and .08 for Anankastia.

* Row 5, Self-Report Anankastia: Correlations with L L M ratings are .12 for Negative Affectivity, .04 for Antagonism, .04 for Detachment, .01 for Disinhibition, and .20 for Anankastia.

The highest convergent validity is seen in Negative Affectivity at .43 [.38, .47].

Navigate back to Figure 1..

### Figure 2. Long description

The matrix displays correlations between Self-Report on the vertical axis and L L M ratings on the horizontal axis. Both axes list the same five domains in order: Negative Affectivity, Antagonism, Detachment, Disinhibition, and Anankastia. Each cell contains a correlation coefficient and a credibility interval in brackets. Darker green indicates higher positive correlation, while light pink indicates near-zero or slightly negative values.

* Row 1, Negative Affectivity (Self-Report): Correlations with L L M domains are .61 [.43, .74] for Negative Affectivity, .25 [.00, .47] for Antagonism, .18 [-.06, .39] for Detachment, .14 [.16, .60] for Disinhibition, and .18 [-.05, .40] for Anankastia.

* Row 2, Antagonism (Self-Report): Correlations are .30 [.05, .51] for Negative Affectivity, .36 [.11, .57] for Antagonism, .25 [.00, .47] for Detachment, .43 [.17, .63] for Disinhibition, and .02 [-.23, .26] for Anankastia.

* Row 3, Detachment (Self-Report): Correlations are .46 [.24, .62] for Negative Affectivity, .16 [-.09, .39] for Antagonism, .39 [.17, .57] for Detachment, .25 [.00, .48] for Disinhibition, and .14 [-.10, .36] for Anankastia.

* Row 4, Disinhibition (Self-Report): Correlations are .24 [.00, .46] for Negative Affectivity, .15 [-.11, .39] for Antagonism, .13 [-.11, .36] for Detachment, .46 [.24, .66] for Disinhibition, and .06 [-.18, .29] for Anankastia.

* Row 5, Anankastia (Self-Report): Correlations are .23 [-.02, .44] for Negative Affectivity, .25 [.00, .48] for Antagonism, .01 [-.23, .26] for Detachment, .14 [-.12, .39] for Disinhibition, and .32 [.08, .52] for Anankastia.

Navigate back to Figure 2..

### Table 1. Long description

The table consists of three columns: Psychopathology domain, Within-person profile similarity, and Between-person profile similarity. The data rows are as follows:

* Negative affectivity: Within-person similarity of .97 and Between-person similarity of .88.

* Antagonism: Within-person similarity of .98 and Between-person similarity of .60.

* Detachment: Within-person similarity of .93 and Between-person similarity of .82.

* Disinhibition: Within-person similarity of .91 and Between-person similarity of .78.

* Anankastia: Within-person similarity of .94 and Between-person similarity of .38.

A note at the bottom explains that coefficients represent correlations between L L M-rated and self-rated domains across 7 within-person and 48 between-person criterion variables.

Navigate back to Table 1..

### Figure 3. Long description

A correlation matrix grid with the Y-axis labeled Clinical Interview Measure and the X-axis labeled Rater. The Y-axis lists eight measures: Negative Affectivity S I D P-I V, Antagonism S I D P-I V, Detachment S I D P-I V, Disinhibition S I D P-I V, Anankastia S I D P-I V, Internalizing S C I D-5, Harmful Substance Use S C I D-5, and Personality Pathology S I D P-I V. The X-axis is divided into five main categories, each with two sub-columns for L L M and Self.

* Negative Affectivity column: Highest correlations are with Internalizing S C I D-5 (L L M .60, Self .55) and Negative Affectivity S I D P-I V (L L M .54, Self .60).

* Antagonism column: Moderate correlations across most measures, peaking at .36 for L L M rating of Negative Affectivity S I D P-I V.

* Detachment column: Shows a strong correlation for Self-ratings with Detachment S I D P-I V (.47) and Internalizing S C I D-5 (.42).

* Disinhibition column: Shows consistent moderate to high correlations across most measures, notably .50 for L L M ratings of Negative Affectivity, Internalizing, and Personality Pathology.

* Anankastia column: Generally lower correlations, with the highest being .30 for L L M rating of Internalizing S C I D-5.

Color coding uses gold for positive correlations and purple for negative correlations, with darker shades indicating higher absolute values. Asterisks indicate credibility intervals that do not contain zero.

Navigate back to Figure 3..

### Table 2. Long description

The table is organized into five rows representing personality domains, each with two sub-rows of examples for High and Low ratings.

1. Negative affectivity:

- High: Quotes describe feeling depressed after a good event and solitary drinking due to feeling bad about life. Rationales cite emotional lability and avoidant coping.

- Low: Quotes describe a baby’s crying as merely unpleasant and excitement about moving. Rationales cite mild distress tolerance and enthusiasm.

2. Antagonism:

- High: Quotes mention wanting to choke someone and wanting things done their way. Rationales cite hostile impulses and entitlement.

- Low: Quotes describe helping a friend study and feeling socially awkward. Rationales cite prosocial behavior and a self-effacing view.

3. Detachment:

- High: Quotes describe forcing oneself to play a game without enjoyment and doing nothing all day. Rationales cite anhedonia and social withdrawal.

- Low: Quotes value spending time with a husband or friends. Rationales cite seeking closeness and intimacy.

4. Disinhibition:

- High: Quotes describe going to a rave instead of sleeping and repeated car mishaps. Rationales cite sensation seeking and irresponsibility.

- Low: Quotes describe moderate social drinking and meeting a professor. Rationales cite controlled behavior and structured commitments.

5. Anankastia:

- High: Quotes describe pushing hard at work without relaxation and starting studying a week early. Rationales cite overconscientiousness and structured planning.

- Low: Quotes describe spontaneous vacation behavior and a relaxed attitude toward late nights. Rationales cite unstructured behavior and lack of preoccupation with order.

Navigate back to Table 2..
