# Supplementary material for: Scalable, context-sensitive psychiatric assessment with large language models and brief diaries
Source: Psychol Med. 2026 Jul 21;56:e231. doi: 10.1017/S0033291726105261 (PMC13439252; doi:10.1017/S0033291726105261)
Supplement: Supplementary file 1 [file S0033291726105261sup001.pdf]

Supplementary materials for "Scalable, context-sensitive psychiatric assessment with large language models and brief audio diaries"

Table S1. LLM scoring procedure

The first step involved transcribing the recordings into text. Video recordings from were first converted to audio-only .mp3 files using ffmpeg, an open-source multimedia processing tool, then audio files were transcribed using the pretrained Whisper Large model, an Automatic Speech Recognition (ASR) model developed by OpenAI.

To score psychopathology from text, we employed six state-of-the-art, commercially available LLMs. These were: OpenAI's GPT-5, Anthropic's Claude-Sonnet-4, Google's Gemini-2.5-Flash-Thinking, xAI's Grok-3, Meta's Llama-4-Maverick and Alibaba's Qwen3-235B. Among these, Llama-4-Maverick and Qwen3-235B are open-source models, while the remaining five are proprietary. Each model was accessed through a unified Python API, leveraging the openai package and OpenRouter API for dynamic model routing. All calls were made with temperature=0 and seed=0. The only exception was Grok-3-Beta, which does not accept seed=0; in this case, the seed was set to 1. For models supporting adjustable reasoning parameters, the reasoning effort was set to "medium".

Our approach was to frame each psychopathology domain from the daily psychopathology inventory as a structured inference task for the LLMs. Each prompt instructed the LLM to estimate the participant's score directly on the target domain based on the daily diary context. The models were instructed to rate each domain on the same 0-100 response scale used in the daily self-report version of the measure and to return only a single numeric value. Responses were programmatically validated, and any output that did not match the required numeric format was discarded and the prompt was re-issued until a valid response was obtained.

For each domain, the model received the following prompt, with the full diary transcript inserted in place of {thoughts}, the target domain inserted in place of {domain}, and the corresponding domain signs inserted in place of {signs}. The signs were defined as follows: Negative Affectivity = anxiety, guilt, shame, irritability, worry, emotional volatility, fear, tendency to experience distressing emotions intensely, or self-criticism; Antagonism = arrogance, hostility, callousness, manipulation, entitlement, grandiosity, interpersonal conflict, exploitation, or lack of empathy; Detachment = social withdrawal, emotional numbness, emotional distance, restricted emotional expression, anhedonia, or a lack of or avoidance of intimacy or connection; Disinhibition = impulsivity, irresponsibility, recklessness, risk taking, poor planning, disregard for consequences, or difficulty delaying gratification; Anankastia = perfectionism, rigid control, preoccupation with rules or order, fear of mistakes, or over-conscientiousness.

LLM Scoring Prompt:

Your task is to assess the participant's level of {domain} based on their daily diary entry describing the most significant event that occurred during their day.

Respond as if you are the participant, using their emotional tone and inner reactions to guide your answer.

Pay attention to signs of {signs}. Use contextual reasoning to determine how strongly the participant likely experienced or expressed {domain}.

Participant's daily diaries:

{thoughts}

Respond with a single number from 0 to 100, where:

- 0 means "not at all"

- 100 means "very much"

Return only the number. Do not include any explanation or punctuation.

The model's response was parsed as a 0-100 numeric rating and this procedure was repeated for each of the five domains, generating a complete set of domain-level LLM-inferred ratings for each diary. The LLM-inferred domain scores were also averaged across the six LLMs to form composite daily domain scores. Code for the LLM scoring procedure is available at: github.com/SripadaLab/audio-diary-llm-assessment

Table S2. Additional information on measures.

Baseline measures

Personality traits were self-reported using the International Personality Item Pool – NEO (IPIP-NEO) – The IPIP-NEO (Johnson, 2014), a short-form, open-source alternative to the flagship assessment of the Five Factor Model of personality—the Revised NEO Personality Inventory (NEO PI-R; Costa & McCrae, 1992). The IPIP-NEO-120 uses 120 items to provide estimates of five NEO domains (Openness, Conscientiousness, Extraversion, Agreeableness, and Neuroticism) and thirty subdomains (six per domain). Items (e.g. "*I get angry easily.*") are rated on a 5-point Likert-style scale from 0-"Very Inaccurate" to 4-"Very Accurate." Domain-level omegas in this study were .83 for Agreeableness, .84 for Openness, .88 for Extraversion, .91 for Neuroticism, and .92 for Conscientiousness.

Interpersonal problems were self-reported using the Inventory of Interpersonal Problems – Short Circumplex (IIP-SC; Soldz, Budman, Demby, & Merry, 1995), a 32-item measure measures difficulties in interpersonal functioning with items (e.g. *"I am too easily persuaded by other people."*) rated from 1-"Not at All" to 5-"Extremely." Octant scores were used in this study.

Adult attachment style was assessed by the Experiences in Close Relationships - Revised (ECR-R; Fraley et al., 2000). The ECR-R is a 36-item measure that assesses individual differences in attachment-related anxiety (i.e., the extent to which people are insecure vs. secure about the availability and responsiveness of romantic partners) and attachment-related avoidance (i.e., the extent to which people are uncomfortable being close to others vs. secure depending on others). Items (e.g., "*I find that my partner(s) don't want to get as close as I would like.*") are rated on from 0-"Strongly Disagree" to 6-"Strongly Agree."

Pathological narcissism was self-reported using the 28-item Brief Pathological Narcissism Inventory (BPNI; Schoenleber et al., 2015). Items (e.g., "*I typically get very angry when I'm unable to get what I want from others*.") are rated on a scale of 0-"Very untrue of me" to 5- "Very true of me." This study used scale scores for narcissistic Grandiosity and Vulnerability

Psychopathology domains were self-reported on the Comprehensive Assessment of Traits Relevant to Personality Disorder (CAT-PD). The CAT-PD (Simms, 2011) includes 216 items and assesses 33 maladaptive personality traits with self-report items rated on a five-point Likert scale (0 – "Very Untrue of Me"; 1 – "Moderately Untrue of Me"; 2 – "Neither True nor Untrue of Me"; 3 – "Moderately True of Me"; 4 – "Very True of Me"). Trait scales were averaged to produce domain-level scores (Ringwald et al., 2024).

Ambulatory assessment procedure and measures

Ecological momentary assessments also typically began the day after baseline assessment. The length of the assessment period was initially set at 21 days but was later shortened to 14 days. Surveys were administered through the MetricWire smartphone application and participants were prompted to answer surveys through push notifications. Surveys were delivered on a randomly initiated schedule. Participants were delivered six surveys per day during an approximately twelve-hour time window that corresponded to the participants' typical waking hours. Blocked random intervals were set so that a minimum of 90 minutes passed between survey and participants were given 20 minutes to initiate a response to each one. A mean number of 68.62 (*SD* = 20.94) surveys were completed per participant.

Participants received ambulatory assessments focusing on momentary measures of affect, stress, and interpersonal interactions since the last assessment. Participants first answered items

assessing current positive and negative affect. Next, participants were asked if anything stressful had occurred since the last assessment. Stress was defined broadly, and participants were instructed to consider whether they themselves felt stressed when the event occurred, not whether they or another person would typically feel stressed in the same situation. If participants reported that the stress involved an interpersonal interaction they reported on their interpersonal behavior during the situation.

Daily stress. Participants reported on stressful events multiple times per day on the momentary surveys. On each momentary survey, participants were asked whether a stressful event occurred since the last assessment. When a stressful event was endorsed, participants were then asked to indicate on a checklist which of the following stressors they experienced: (1) an argument/disagreement, (2) social disappointment/let-down, (3) work/school-related event, (4) home-related event, (5) health-related issue, (6) vicarious stress from a close friend/relative, and (7) other stress. If they endorsed experiencing more than one event, they were asked "Out of all the stressors you selected, which one is MOST stressful?" and answered the additional questions about that event. For this study, we used participant reports of how stressful the event was when it happened (i.e., severity). Severity of the stressor was rated on a sliding scale from 0 – "not at all" to 100 – "extremely."

We used the event checklists and stress severity ratings to calculate two stress outcomes per day: (1) sum of perceived stressful events, indexed by the sum of events endorsed on a day and (2) perceived *severity* of stressful events, indexed by the mean of severity ratings on a day.

Positive and negative affect. Affect was self-reported measured using ten adjectives in the randomly prompted surveys. Positive affect was represented by *Happy*, *Proud*, *Content*, *Excited*, and *Relaxed*. Negative affect was represented by *Ashamed*, *Nervous*, *Hostile*, *Sad*, and

Angry. Participants were asked questions in the form *"How ADJECTIVE do you feel right now?"* Ratings were made on a scale from 0 - "Not at All" to 100 –"Extremely."

Dominant and warm behavior. Interpersonal behavior was self-reported by the Visual Interpersonal Analogue Scale (VIAS; Woods et al., 2023). Dominant behavior was assessed using a visual analogue slider bar ranging from -50 – "Accommodating/Submissive/Timid" to 50 – "Assertive/Dominant/Controlling." Affiliative behavior was rated on a similar visual slider bar ranging from -50 – "Cold/Distant/Hostile" to 50 – "Warm/Friendly/Caring".

Impulsivity. At each survey, participants were rated the question "How would you describe your behavior right now" on a sliding scale from 0 – "In control" to 100 – "Impulsive."

Table S3a. Correlations between daily LLM-ratings and baseline self-reports of psychopathology

CAT-PD \ LLM	Negative Affectivity	Antagonism	Detachment	Disinhibition	Anankastia
Negative Affectivity	.48	.25	.24	.35	.13
Antagonism	-.08	.17	.19	.39	-.20
Detachment	.31	.07	.33	-.07	.22
Disinhibition	.27	.04	.13	.24	-.03
Anankastia	.04	.12	.07	.08	.18

Table S3b. Correlations between daily self-reports and baseline self-reports of psychopathology

CAT-PD \ DAILY SELF-REPORT	Negative Affectivity	Antagonism	Detachment	Disinhibition	Anankastia
Negative Affectivity	.72	.40	.61	.42	.20
Antagonism	.19	.37	.15	.44	.12
Detachment	.18	.08	.47	-.10	-.02
Disinhibition	.43	.23	.45	.39	-.24
Anankastia	.17	.16	.12	.08	.59

Table S4. Profile of associations with external criterion at the within-person level.

	Neg. Affectivity		Antagonism		Detachment		Disinhibition		Anankastia	
	LLM	Self	LLM	Self	LLM	Self	LLM	Self	LLM	Self
Positive Affect	-.30	-.37	-.12	-.11	-.18	-.30	-.06	-.06	-.13	-.08
Negative Affect	.30	.48	.14	.24	.09	.32	.09	.18	.09	.17
Impulsivity	.19	.21	.11	.15	.07	.13	.09	.16	.09	.08
Dominance	-.03	-.05	.06	.09	.00	-.06	-.01	.00	-.05	-.06
Warmth	-.24	-.19	-.23	-.23	-.15	-.22	-.08	-.09	-.10	-.05
Sum of Stressors	.21	.20	.10	.09	.08	.12	.04	.05	.11	.14
Severity of Stressors	.18	.17	.08	.14	-.02	.09	.10	.06	.02	.04

Table S5. Profile of associations with external criterion at the between-person level.

		Neg. Affectivity		Antagonism		Detachment		Disinhibition		Anankastia	
		LLM	Self	LLM	Self	LLM	Self	LLM	Self	LLM	Self
Neuroticism (IPIP-NEO)	Anxiety	.43	.59	.11	.19	.11	.48	.13	.24	.30	.17
	Anger	.25	.27	.26	.29	.25	.23	.25	.22	.16	.06
	Depression	.52	.70	.15	.27	.21	.67	.26	.41	.21	.15
	Self-Consciousness	.31	.29	.15	.08	.27	.38	.06	.02	.24	-.04
	Immoderation	.15	.39	-.07	.20	-.02	.27	.16	.41	.03	.00
	Vulnerability	.50	.60	.18	.25	.13	.51	.21	.26	.32	.12
Agreeableness (IPIP-NEO)	Trust	-.19	-.35	-.23	-.28	-.38	-.38	-.06	-.18	.08	.01
	Morality	.21	-.12	.16	-.20	-.03	-.09	-.07	-.30	.31	.00
	Altruism	-.05	.01	-.06	-.07	-.28	-.23	-.10	-.06	.01	.05
	Cooperation	-.06	-.21	-.28	-.45	-.27	-.21	-.30	-.29	.11	-.07
	Modesty	.28	.14	.07	-.13	-.02	.16	-.02	-.15	.22	-.02
	Sympathy	.18	.19	-.07	-.04	-.07	.03	-.01	.11	.20	-.02
	Friendliness	-.26	-.29	-.12	-.18	-.38	-.46	-.04	-.07	-.13	-.03
Extraversion (IPIP-NEO)	Gregariousness	-.26	-.20	-.11	-.09	-.40	-.42	.06	.02	-.13	-.02
	Assertiveness	-.31	-.34	-.08	-.11	-.17	-.40	-.07	-.10	-.13	.07
	Activity Level	-.06	.07	.14	.26	-.05	.01	.10	.10	.10	.52
	Excitement Seeking	-.23	.02	.08	.16	-.06	-.11	.26	.31	-.34	-.09
	Cheerfulness	-.36	-.43	-.08	-.15	-.29	-.56	-.06	-.08	-.24	-.01
	Self-Efficacy	-.21	-.33	-.01	-.18	-.09	-.38	-.13	-.36	-.15	-.01
Conscientiousness (IPIP-NEO)	Orderliness	-.05	-.12	.04	-.10	-.01	-.10	-.04	-.19	.03	.10
	Dutifulness	.13	-.11	.09	-.18	-.07	-.21	-.07	-.40	.26	.17
	Achievement Striving	-.02	-.15	.03	-.05	-.15	-.19	-.18	-.27	.16	.29
	Self-Discipline	-.22	-.33	.04	-.13	-.22	-.41	-.16	-.39	-.10	.10
	Cautiousness	-.22	-.33	.04	-.13	-.22	-.41	-.16	-.39	-.10	.10
Openness (IPIP-NEO)	Imagination	.06	.26	.00	.15	.11	.17	.07	.30	.03	.12
	Artistic Interests	-.07	-.10	-.01	-.10	-.06	-.32	.00	.06	.00	.01
	Emotionality	.07	.20	-.04	.04	-.32	-.10	.09	.17	-.01	.12
	Adventurousness	-.25	-.14	-.15	-.07	-.20	-.33	.05	.11	-.25	-.16
	Intellect	-.10	-.05	-.07	-.01	.02	-.20	-.09	.04	.04	.11
	Liberalism	-.01	.09	-.12	-.12	-.07	-.08	-.04	.18	-.08	-.13
Interpersonal problems (IIP-SC)	Domineering	.10	.31	.11	.31	.32	.30	.16	.27	.03	.15
	Vindictive	.23	.37	.17	.11	-.02	.08	.27	.29	.04	.08
	Cold	.31	.47	.11	.14	-.04	.37	.10	.29	.22	.23
	Socially Avoidant	.22	.43	.06	.07	.05	.38	.08	.31	.20	.14
	Nonassertive	.24	.40	.04	.15	.10	.45	.09	.29	.22	.14
	Exploitable	.35	.41	.08	.19	.27	.52	.07	.16	.22	.05
	Overly Nurturant	.24	.39	.18	.29	.31	.57	.12	.24	.11	.15
	Intrusive	.14	.38	.20	.37	.28	.43	.19	.27	-.02	.11
Attachment style (ECR-R)	Avoidance	.12	.06	.13	.09	.18	.22	.09	.10	-.01	-.21
	Anxiety	.21	.24	.06	.00	.11	.26	.11	.16	.04	-.16
Narcissism (BPNI)	Vulnerability	.24	.57	.13	.35	.12	.53	.24	.49	.06	.28
	Grandiosity	.00	.37	-.02	.26	-.06	.26	.21	.48	-.04	.33
EMA Variables	Positive Affect	-.33	-.34	.07	-.09	-.27	-.36	.05	-.09	-.26	.06
	Negative Affect	.49	.83	.12	.51	.13	.68	.21	.54	.21	.33
	Impulsivity	.23	.60	-.07	.43	-.05	.50	.29	.64	.16	.24
	Dominance	.06	-.22	.42	.10	-.18	-.28	.32	-.12	-.06	-.18
	Warmth	-.27	-.17	-.32	-.27	-.40	-.26	-.30	-.03	-.11	-.10
	Sum of Stressors	.41	.28	.14	.17	-.04	.24	.14	.16	.29	.13
	Severity of Stressors	.34	.47	.19	.24	.12	.37	.30	.33	-.03	.12
